# Is Increasing Coal Seam Gas Well Development Activity Associated with Increasing Hospitalisation Rates in Queensland, Australia? An Exploratory Analysis 1995–2011

**DOI:** 10.3390/ijerph14050540

**Published:** 2017-05-18

**Authors:** Angela K. Werner, Cate M. Cameron, Kerrianne Watt, Sue Vink, Paul Jagals, Andrew Page

**Affiliations:** 1 Sustainable Minerals Institute, The University of Queensland, St. Lucia, QLD 4072, Australia; s.vink@smi.uq.edu.au; 2 Menzies Health Institute Queensland, Griffith University, Logan, QLD 4131, Australia; cate.cameron@griffith.edu.au; 3 College of Public Health, Medical and Veterinary Sciences, James Cook University, Townsville, QLD 4811, Australia; kerrianne.watt@jcu.edu.au; 4 Children’s Health and Environment Programme, University of Queensland, Centre for Children’s Health Research, Brisbane, QLD 4101, Australia; p.jagals@uq.edu.au; 5 Centre for Health Research, Western Sydney University, Penrith, NSW 2150, Australia; a.page@westernsydney.edu.au

**Keywords:** coal seam gas, environmental health, hospital admissions, Queensland, time series, unconventional natural gas

## Abstract

The majority of Australia’s coal seam gas (CSG) reserves are in Queensland, where the industry has expanded rapidly in recent years. Despite concerns, health data have not been examined alongside CSG development. This study examined hospitalisation rates as a function of CSG development activity in Queensland, during the period 1995–2011. Admissions data were examined with CSG well numbers, which served as a proxy for CSG development activity. Time series models were used to assess changes in hospitalisation rates for periods of “low”, “medium”, “high”, and “intense” activity compared to a period of “very low” activity, adjusting for covariates. “All-cause” hospitalisation rates increased monotonically with increasing gas well development activity in females (324.0 to 390.3 per 1000 persons) and males (294.2 to 335.4 per 1000 persons). Hospitalisation rates for “Blood/immune” conditions generally increased for both sexes. Female and male hospitalisation rates for “Circulatory” conditions decreased with increasing CSG activity. Hospitalisation rates were generally low for reproductive and birth outcomes; no clear associations were observed. This study showed some outcomes were associated with increasing CSG development activity. However, as a condition of data access, the population and outcomes were aggregated to a broad geographic study area rather than using higher geographic resolution data. Higher resolution data, as well as other data sources, should be explored. Further research should be conducted with an expanded time period to determine if these trends continue as the industry grows.

## 1. Introduction

Coal seam gas (CSG), shale gas, and tight gas are all classified as unconventional natural gas, which are extracted from low permeability source rock, requiring additional technology and a greater number of wells to release the gas [1]. CSG is comprised mostly of methane and is held in place by water pressure in the natural fractures (i.e., cleats) of coal seams. Dewatering reduces the water pressure, allowing gas to flow to the surface, reducing the need for hydraulic fracturing in many CSG wells [2,3,4,5]. This differs from other forms of unconventional natural gas development (UNGD), such as shale gas, where permeable fractures must be created (i.e., through hydraulic fracturing) to allow the gas to flow [3]. Chemicals are used or produced throughout UNGD processes, and there are established mechanisms for which these chemicals can be released to environmental media [6]. Pollutants as by-products of these processes have known or potential human health impacts, with primary exposures being inhalation or ingestion [7].

CSG has been extracted in Queensland for nearly 30 years, but the industry has grown markedly in the last decade due to an increasing local and international demand for liquefied natural gas (LNG) [8]. The expansion of the CSG industry will see Australia as a top exporter of LNG by 2020 [9]. With the growth of the CSG industry, residents have raised concerns about potential environmental and health impacts [3,5,9,10,11,12,13,14,15,16]. Some residents living in CSG areas in New South Wales and Queensland have reported health impacts that they have experienced [17], but this has not been investigated using epidemiologic studies [18]. 

Many studies have addressed environmental health impacts associated with other forms of UNGD, with the focus typically on shale gas, for which the extraction process is more chemical intensive than for CSG [18]. Because of these and other differences between the development and extraction of these two resources (e.g., geological formations, extraction, processes, regulatory frameworks), outcomes from shale gas studies may not be readily extrapolated to the CSG context [10,19]. Therefore, whilst broader UNGD studies have to be used here to provide context for potential health impacts and for study findings, it is important to conduct studies focused on CSG. This was the purpose of the present exploratory study. 

In previous work conducted by the authors, hospital admission rates in a CSG area were compared to two study areas (coal mining and rural/agricultural) over time. Rates increased for some health outcomes (e.g., “Blood/immune” and “Respiratory”) and age groups (e.g., 0–4, 5–9, 10–14, and 20–34 year old) in the CSG area compared to the other study areas [19,20,21]. This paper focuses solely on the CSG study area to examine whether changes in hospitalisation rates for residents of this area vary over time as a function of CSG development activity (e.g., construction, drilling of wells, etc. prior to extraction).

Particular focus is given to those primary diagnosis codes that relate to health outcomes previously shown in CSG-specific or broader UNGD studies to be potentially associated with CSG exposure: “Blood/immune” [19], “Circulatory” [22], “Respiratory” [23,24,25,26], and “Perinatal” and “Congenital” outcomes [27,28,29,30].

## 2. Materials and Methods 

The University of Queensland Human Research Ethics Committee approved the ethics application (number 2012000582) and Queensland Health provided access to confidential data through the Public Health Act (number RD004515).

### 2.1. Study Area

**Figure 1:**
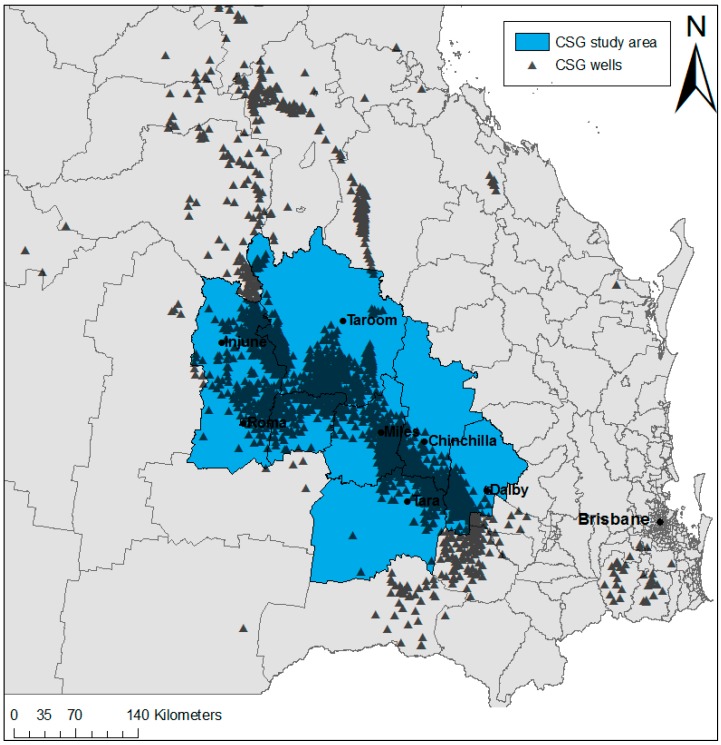
The coal seam gas (CSG) study area, depicting all statistical local area (SLA) boundaries and CSG well locations in Queensland, Australia.

The CSG setting, corresponding geographic study area, and study time period have been described in detail elsewhere [19]. Briefly, an area was selected based on a strong, contemporary CSG industry presence in a region where the primary land use activity has historically been agriculture with a minimum of other energy resource extraction activity. The area was selected using “MinesOnlineMaps” [31], which provides information on CSG well locations at the time of site selection (2011) for the entire study period (1995–2011). Figure 1 shows the CSG study area, including CSG well locations. 

Statistical Local Areas (SLAs) are general use spatial units used to collect statistics across Australia, and each SLA covers a distinct area [32]. SLAs were combined to facilitate access to health data; therefore, the CSG study area comprises nine SLAs, and hospital admissions were aggregated to this broad area.

### 2.2. Data

Hospital admissions data were provided by Queensland Health through the Queensland Hospital Admitted Patient Data Collection (QHAPDC). Primary diagnoses via International Classification of Diseases (ICD) codes were obtained for each admission for each calendar year between 1995–2011, along with age, sex, and date of admission. Data were obtained only on residents of the CSG study area and did not include travellers or workers who resided elsewhere. ICD-9 Clinical Modification (ICD-9-CM) codes were forward-mapped to ICD-10 Australian Modification (ICD-10-AM) codes for uniformity. Selected ICD chapters were analysed based on a-priori hypotheses of the potential impact of CSG development, including “All-cause”, “Diseases of the blood and blood-forming organs and certain disorders involving the immune mechanism” (ICD-10 chapter III, code range D50–D89; ICD-9 chapter 3, code range 280–289), “Diseases of the circulatory system” (ICD-10 chapter IX, code range I00-I09; ICD-9 chapter 7, code range 390–459), “Diseases of the respiratory system” (ICD-10 chapter X, code range J00–J99; ICD-9 chapter 8, code range 460–519), “Certain conditions originating in the perinatal period” (ICD-10 chapter XVI, code range P00–P96; ICD-9 chapter 15, code range 760–779), and “Congenital malformations, deformations and chromosomal abnormalities” (ICD-10 chapter XVII, code range Q00–Q99; ICD-9 chapter 14, code range 740–759). Further detail regarding QHAPDC data are provided elsewhere [19]. 

Population data (resident population by sex and age group) were obtained from the Australian Bureau of Statistics (ABS) for each calendar year [19]. Yearly population data were divided by 12 to obtain monthly estimated resident population data. Data were then grouped according to broader age groups (i.e., 0–19 years, 20–64 years, 65–85+ years) for use in the adjusted models.

Covariate data were collected from the ABS for Census years (every five years) from 1991 onward. Data included the number of persons Australian-born, employed full-time, Indigenous, and in white collar occupations, as well as the median household income and mean household size, with weighted interpolation used to estimate data for intercensal years [19]. Covariate data were aggregated to the same level (SLAs) as hospital admissions data to ensure consistency. Hence, all data were aggregated to the nine SLAs that make up the study area.

CSG well data were obtained from the Queensland Government [31]. Data were collected as number of wells within each SLA in the study area. Cumulative number of wells were calculated for each month of CSG development for the entire CSG area over the study time period. The number of wells was considered to be an indicator of CSG development activity and served as a proxy for these activities. Such activities included construction activities, pad preparation, well drilling, and well completion [23,33]. 

**Figure 2:**
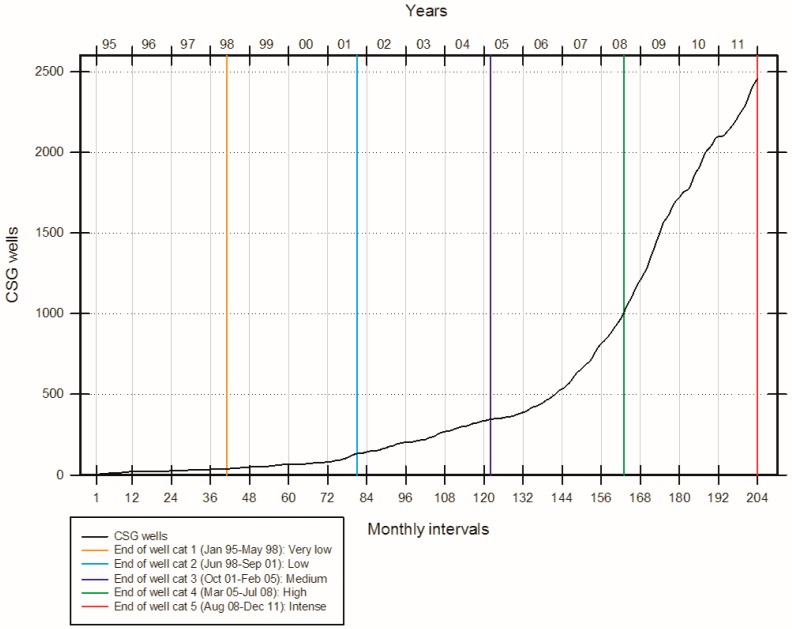
Monthly number of coal seam gas (CSG) wells and corresponding well categories, 1995–2011. Note: the “very low” period of activity (January 1995–May 1998) served as the reference category.

A well category variable was created to divide the gas well development (GWD) activity into quintiles (i.e., “very low”, “low”, “medium”, “high”, “intense”). Figure 2 shows the CSG well numbers and the GWD categories used in the analyses. GWD activity was categorised as follows: “very low” = January 1995–May 1998; “low” = June 1998–September 2001; “medium” = October 2001–February 2005; “high” = March 2005–July 2008; and “intense” = August 2008–December 2011. The “very low” category served as the referent. 

### 2.3. Analyses

A series of interrupted time series regression models were conducted, stratified by sex, to investigate the association between CSG development activity (categorised in quintiles of activity, representing periods of lowest to highest CSG development activity) and monthly hospitalisation rates (for 204 months, covering the period 1995–2011). Time series regression models were specified to account for autocorrelation (in this instance, seasonal effects), random variation, and trend (or “drift”) that are inherent in time series data [34]. Regression models were also adjusted for age (0–19, 20–64, and 65+ years) and the proportion of Australian-born, employed full-time, Indigenous, white collar occupations, median household income, and mean household size, specified as continuous variables. Statistical analyses were completed using PROC ARIMA in SAS 9.4 (SAS Institute Inc., Cary, NC, USA).

## 3. Results

In the period 1995–2011, there were 238,457 admissions to hospital for the CSG study area. The minimum number of admissions in one month was 718, and the maximum number of admissions in one month was 1610. The GWD category defined as “very low” had the fewest hospital admissions (n = 40,654). The remaining GWD category admissions were: 43,511 admissions for the “low” category; 46,577 for the “medium” GWD activity category; 51,492 for the “high” category; and 56,223 admissions for the “intense” GWD category. Increases in the number of admissions were not uniform across diseases (i.e., ICD chapters) or age groups.

Table 1 and Table 2 show the results for the adjusted and unadjusted models for females and males, respectively. For females, “All-cause” and “Congenital” hospitalisation rates decreased slightly after adjusting for covariates. “Blood/immune”, “Circulatory”, and “Respiratory” disease admission rates all increased after adjusting for covariates. “Perinatal”-related admission rates stayed the same or varied slightly after adjustment. For males, “All-cause” and “Perinatal” hospitalisation rates decreased after adjustment, “Blood/immune”, “Respiratory”, and “Congenital” disease-related admission rates increased, and “Circulatory” disease-related rates varied after adjusting for covariates. The remainder of the results focuses on the adjusted rates. 

**Figure 3:**
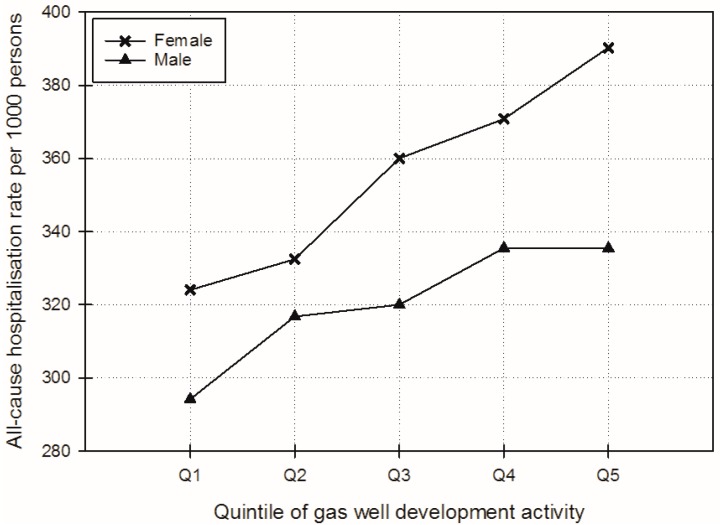
Adjusted female and male “All-cause” hospitalisation rates per 1000 persons over gas well development activity quintiles (adjusted for: age; proportion Australian-born; employed full-time; Indigenous; white collar (managerial, administrative, professional); weighted average of median household income; and weighted average of mean household size).

**Figure 4:**
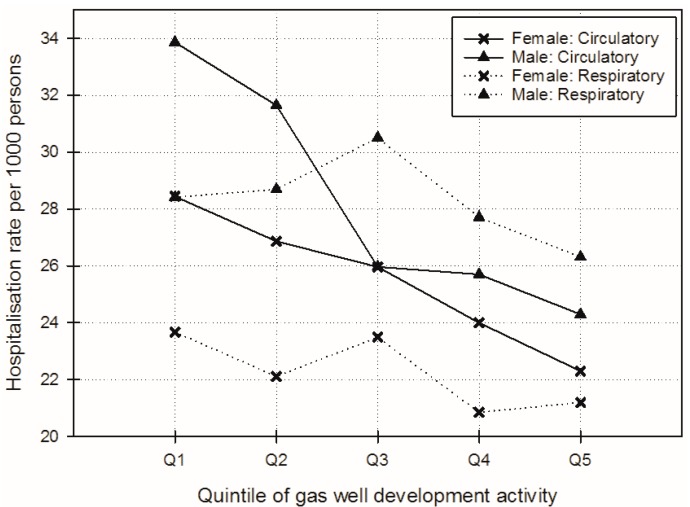
Adjusted female and male “Circulatory” and “Respiratory” hospitalisation rates per 1000 persons over gas well development activity quintiles (adjusted for: age; proportion Australian-born; employed full-time; Indigenous; white collar (managerial, administrative, professional); weighted average of median household income; and weighted average of mean household size). Note there are scale differences across figures as rates for individual chapters are lower than those for “All-cause” admissions.

Female “All-cause” hospitalisations rates increased monotonically with increasing categories of GWD activity from 324.0 to 390.3 per 1000 persons. Male “All-cause” rates also increased (from 294.2 to 335.4 per 1000 persons) but with less magnitude (see Figure 3). For the outcomes that were the focus of the a-priori hypotheses for this study, the highest rates for both females and males were for “Circulatory” and “Respiratory”-related diseases. For “Circulatory” diseases, hospitalisation rates decreased as GWD increased, both for females (from 28.4 to 22.3 per 1000 persons) and for males (from 33.9 to 24.3 per 1000 persons for “Circulatory”) (Figure 4). “Respiratory”-related disease admission rates showed fluctuation but a general slight decreasing trend (from 23.7 to 21.2 per 1000 persons for females and from 28.4 to 26.3 per 1000 persons for males). 

**Figure 5:**
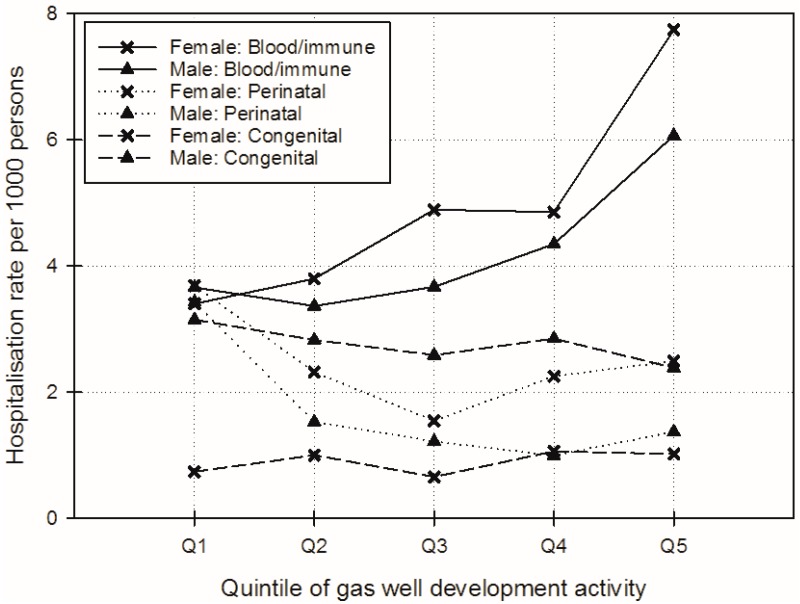
Adjusted female and male “Blood/immune”, “Perinatal”, and “Congenital” hospitalisation rates per 1000 persons over gas well development activity quintiles (adjusted for: age; proportion Australian-born; employed full-time; Indigenous; white collar (managerial, administrative, professional); weighted average of median household income; and weighted average of mean household size).

“Blood/immune” disease hospitalisation rates increased with higher levels of GWD activity (compared to lower levels), with an increasing trend in females (from 3.4 to 7.7 per 1000 persons) and males (from 3.7 to 6.1 per 1000 persons) (see Figure 5). There was a slight inverse trend between “Perinatal” and “Congenital” hospitalisation rates and increased levels of GWD activity (Figure 5). There were also fewer admissions for health conditions covered by these ICD chapters compared to other chapters analysed here (861 and 1074 “Perinatal” admissions for females and males, respectively, and 437 and 742 “Congenital” admissions for females and males, respectively).

Associations between levels of GWD activity and all other ICD chapters were also investigated (Supplementary Tables S1 and S2). Monotonic increases were evident in females for “Infectious disease”, “Endocrine”, and “Injuries” after adjustment for covariates (Supplementary Table S1). 

## 4. Discussion

This study investigated the association between CSG well development and hospitalisation rates in Queensland (Australia), based on previous studies suggesting that the presence of CSG development may have environmental and/or health effects, as well as the recent prominence of the expansion of CSG extraction in Australia in public and political discourse. This study found equivocal associations between hospitalisation rates and gas well development activity. Whilst a clear dose-response association was found between “All-cause” hospital admissions and increasing gas well development, associations between specific primary diagnosis groups were less clear. 

The lack of CSG-specific studies requires examining the literature more broadly, using the limited number of UNGD-related studies for context. Studies focusing on potential human health effects related to UNGD have employed several study designs, including case-control, cross-sectional, ecological, nested case-control, and retrospective cohort designs. Results from an ecological study noted associations between “Cardiology” admissions and number and density of wells [22]. Some studies reported “Respiratory”-related findings. One of the few, if only, studies to use a nested case-control design found an association between asthma exacerbations and levels of UNGD activity [25], whilst self-reported upper respiratory symptoms were correlated with distance from UNGD activity in a cross-sectional study [24]. Other associations, including preterm birth and high-risk pregnancy and UNGD activity exposure (via inverse distance-squared metric) [29], congenital heart defects and neural tube defects and UNGD activity exposure (via inverse distance weighted (IDW) well count) [28], and low birth weight and small for gestational age and UNGD activity exposure (via IDW well count) [27] were also reported in several retrospective cohort studies. 

Similar associations were not observed in the present ecological study. For “Circulatory”, “Respiratory”, “Perinatal”, and “Congenital” outcomes, associations between hospital admissions and gas well development were not evident or were associated with decreases in admissions over time. However, it is important to note that this study examined aggregations of multiple outcomes in broad disease groups (i.e., ICD chapters) rather than specific diseases (i.e., ICD codes); therefore, the findings of this study are not directly comparable to previous studies where specific outcomes have been investigated. Differences in study design may also contribute to these differences, including the need to use a geographically large dataset for this study.

This is the first study investigating the potential health impacts of increasing CSG development in Queensland by using an objective outcome measure (hospital admissions), and over a period comprising the lifespan of the CSG industry development in the area from its inception, exploration, and intensive development in the most recent period. There are very few studies that have examined any form of UNGD and associated changes in hospital admissions. Studies from the United States have analysed hospital admissions data over 5-, 6.25-, and 13-year study periods [22,35,36] whereas this study used a 17-year study period with over 235,000 admissions. While the findings from those studies cannot be directly extrapolated to the CSG context due to differences between CSG, shale gas, and tight gas [10,19], studies that can be categorised as UNGD are predominantly discussed here due to the dearth of available CSG-specific studies.

A previous ecological study compared all-age hospitalisation rates in the same CSG study area used here to coal mining and rural/agricultural study areas and found that hospitalisation rates for “Blood/immune” diseases, “Neoplasms”, and “Congenital” outcomes increased in the CSG study area relative to the hospitalisation rates in the other study areas [19,37]. Additionally, age-specific studies found increasing “Blood/immune” disease admission rates in a CSG study area compared to the other study areas and decreasing rates for one child/adolescent age group [20,21]. 

“Neoplasms” were not considered in the a-priori hypotheses for the current study because of the time period of CSG development and consideration of lag times [19]. In the current study, only “Blood/immune” disease admissions showed generally increasing rates with increasing GWD activity. Other studies that examined hospital admissions concluded there were increasing admission rates in areas with UNGD compared to other areas [35] or that there was no association between these admissions and well numbers or density [22]. One of these studies [35] compared outcomes at the county level, where there were varying levels of UNGD (and is not peer-reviewed), while the other employed a study design similar to that used here.

Increases in cardiology-related outcomes have been reported previously [22,36]. However, in this study, decreased hospital admissions due to “Circulatory” conditions (which encompass cardiology outcomes) with increasing GWD activity periods were observed. These differences could be because specific outcomes were examined in previous studies rather than all “Circulatory” admissions in the present study because of differences in UNGD activity measures and exposure metrics or previously mentioned differences in study design. However, more research is needed to determine the extent to which findings are consistent with specific outcomes and different CSG measures (e.g., number or density of wells rather than GWD activity periods).

A hazard ranking methodology has been used previously to assess potential impacts of UNGD in the United States, and the authors concluded there was a high concern for negative impacts on public health due to UNGD-related changes in air quality [38]. In examining self-reported respiratory symptoms, an association was found between upper respiratory symptoms and UNGD activity, but not for lower respiratory symptoms [24]. Other studies have noted associations between respiratory-related outcomes (e.g., asthma, chronic obstructive pulmonary disease, pneumonia, upper respiratory infection) and shale gas development activity [25,36]. However, the strength of evidence of these studies is variable. One of these studies accounted for individual-level exposures and outcomes using a nested case-control design [25] and the other was not peer-reviewed. Scientific quality also varied in studies where no association was observed [22,35]. In the present study, “Respiratory” disease admission rates only increased slightly for males through the “medium” GWD activity period, then decreased, while female rates varied over time. 

One possible explanation for the different findings is that the data used in this study represent serious morbidity, as the data relate to residents admitted to hospital (for 24 h or more) rather than self-report of symptoms. It is possible that respiratory symptoms could be due to airborne irritant exposures related to UNGD, including those associated with flaring and diesel exhaust [24], but residents may not present to hospital, so would not have been detected in this study. The difference in study designs (e.g., nested case-control versus ecological) and exposure metrics (e.g., individual-level for development activity metrics versus broad exposure classification) may also contribute to the differences in findings and differences in internal validity. Furthermore, there can be age-specific differences for these conditions, which would require examining admissions by age group. 

UNGD activity and reproductive and birth outcomes have been investigated in several studies, with most using retrospective cohort designs focusing on shale or tight gas activity in Pennsylvania or Colorado. Associations have been noted for congenital heart defects, high-risk pregnancy, low birth weight, neural tube defects, prematurity, reduced APGAR scores, and small for gestational age [27,28,29,39]. One study found an association between neonatology inpatient rates and number of wells [22]. Inverse associations have been reported between UNGD activity and low birth weight and prematurity [27,28]. One study used time series analyses and noted no associations between birth defects and levels of UNGD activity or in areas with and without UNGD in Pennsylvania [40]. For these reasons, “Perinatal” and “Congenital” admissions were included in the present study; no clear increases or decreases with increasing GWD activity were observed. However, there were few admissions over the study period for these chapters, resulting in very low rates. Queensland Health maintains a Perinatal Data Collection, which may be more useful to explore for reproductive and birth outcomes. 

A limitation of this study is the use of a broad geographic study area to represent the CSG area rather than using a higher geographic resolution corresponding to individual SLAs or postal codes. Aggregation of the data to the whole population was a condition of access to hospital data by primary diagnosis and was stipulated as part of Queensland Health approvals. The result was that the use of a large geographic study area limited the ability to consider geographic and temporal variation in the data. As such, additional analyses, like using patient distance from CSG infrastructure as an individual proxy measure of exposure to CSG development activities, could not be included in the present analysis. 

A geographically large area also precludes the evaluation of population effects that may be occurring in a specific area within the broader study area. As previously mentioned, aggregation of health outcomes into broad disease groups (represented by ICD chapters) did not allow for examination of ICD codes for specific diseases (e.g., asthma), so the results are not directly comparable to studies, which may have employed different study designs, where these outcomes were examined. The lack of CSG-specific studies that have examined potential health impacts means that the findings had to be evaluated in a broader context, but this study adds to the limited literature base.

It is possible that the rates that are presented here (i.e., using QHAPDC hospitalisation data) were an underestimate of serious morbidity in the community due to the lack of data on residents not seeking hospital care and data on General Practitioner visits. It would be beneficial to capture these data where residents might present with symptoms but not be admitted to hospital. Such data could then be assessed and compared with the hospital admission findings. Changes in rates could in part be due to broader changes in health care provision resulting in shorter lengths of stay and increased re-admissions. Changes in rates could also be due to changes in other stressors for residents living in this area, which could impact hospitalisation rates over time for the study period. However, previous work that compared admissions in the CSG area to two other geographic areas (non-CSG) found admission rates in the CSG area increased for certain outcomes above rates in other areas for the same period [19]. 

The GWD variable was not a highly sensitive measure as it assumes that all individuals in the area had the same level of exposure. Post-2011, CSG-related activity (particularly construction) continued to rise rapidly. Additional analyses of data post-2011 is important to determine whether these trends continue. Additionally, the CSG area experienced extended drought, major floods and reconstruction activities, such as the construction of the Kogan Creek power station, and migration of young adults into the area as a result of employment. Potential confounders were included in the analyses to account for changes over time in key sociodemographics (such as age, socioeconomic status, Indigenous status), but it is unclear how other environmental and community development factors might have affected hospital admission trends. 

Additional research should consider these findings in light of the expanding CSG industry, with the projection of up to 40,000 wells to be drilled by 2030 [8,9]. Ideally, higher resolution data should be used so a resident’s location can be examined in conjunction with CSG and health outcomes data (i.e., at the individual level rather than at the broad geographic level with an ecological exposure metric in the present study, which may obscure results). This would also allow for more in-depth exploration of specific diseases (i.e., through ICD codes).

The present study adjusted for age; however, the inconsistency of admission increases across age groups and diseases suggest that age- and disease-specific groups warrant further investigation. Future work should also focus on gender-specific differences in the health impacts of CSG development. In this study, there were monotonic increases in hospitalisation rates in several disease categories (i.e., ICD chapters) among females but not for males. To our knowledge, no previous studies have explored gender-specific hospitalisation rate differences in detail, so it is not possible to determine whether the observed differences are due to gender-specific differences in the health impacts of CSG development or if they reflect gender-specific hospitalisation rates per se. Notably, increases (though not monotonic) in hospitalisation rates were observed in some disease categories (e.g., “Neoplasms”, “Infectious disease”, “Nervous system”, and “Injury”) that were not included in the a-priori hypotheses. These conditions should be explored in more detail in future studies, with more sophisticated study designs to allow for conclusions about causality whilst minimising bias.

Investigating associations between health impacts and CSG development requires consideration of cumulative impacts. Brown et al. [41] concluded that residents living near UNGD are exposed to various air pollutants at varying intensities over time. These exposures, as well as additional exposures such as water pollutants, should be taken into account in future studies so residents can better understand local health impacts, healthcare providers can better identify and evaluate symptoms related to such exposures, and policymakers can better understand mitigation measures that can be taken to reduce exposures.

## 5. Conclusions

There were increasing rates of hospitalisation for certain health outcomes occurring contemporaneously with increasing CSG development. These associations were generally inconsistent with previous literature, which may be due to differences in study designs and specific diseases that were examined. The increasing intensity of CSG well development activity in Queensland may have resulted in population health impacts, as measured by hospital admissions. The findings presented here add to the previously identified lack of CSG-specific studies on health impacts in populations. Higher resolution data and specific outcomes, such as reproductive and birth outcomes, should be examined in future studies. Data available after the current study period (post-2011) should also be analysed to see if similar trends continue.

## Figures and Tables

**Table 1:** Adjusted and unadjusted female all-age hospitalisation rates (per 1000 persons), 95% CI, and significance for trend across gas well development activity quintiles in a coal seam gas area in Queensland, Australia ^1^.

	Very Low (Jan. 1995–May 1998)	Low (Jun. 1998–Sep. 2001)	Medium (Oct. 2001–Feb. 2005)	High (Mar. 2005–Jul. 2008)	Intense (Aug. 2008–Dec. 2011)	*p*-Value for Linear Trend
*Adjusted*						
All-cause	324.0 (295.3–352.8)	332.5 (312.6–352.3)	360.0 (333.8–386.3)	370.8 (338.5–403.1)	390.3 (355.3–425.3)	0.0003
Blood/immune	3.4 (0.9–5.9)	3.8 (2.5–5.1)	4.9 (2.9–6.9)	4.9 (2.3–7.4)	7.7 (5.1–10.4)	0.0009
Circulatory	28.4 (22.7–34.2)	26.9 (23.7–30.1)	26.0 (21.3–30.6)	24.0 (18.0–30.0)	22.3 (16.1–28.5)	0.0443
Respiratory	23.7 (17.8–30.0)	22.1 (17.8–26.4)	23.5 (17.9–29.1)	20.8 (14.2–27.5)	21.2 (13.8–28.6)	0.5146
Perinatal	3.7 (2.3–5.1)	2.3 (1.7–3.0)	1.5 (0.5–2.6)	2.2 (0.9–3.6)	2.5 (1.1–3.8)	0.0207
Congenital	0.7 (0.0–1.5)	1.0 (0.6–1.4)	0.7 (0.1–1.2)	1.1 (0.3–1.8)	1.0 (0.3–1.8)	0.4174
*Unadjusted*						
All-cause	328.5 (311.9–345.1)	340.7 (320.0–361.4)	363.7 (340.5–386.9)	382.4 (358.6–406.1)	409.2 (384.8–433.7)	<0.0001
Blood/immune	2.0 (1.1–3.0)	2.4 (1.1–3.7)	3.1 (1.7–4.4)	3.4 (2.1–4.8)	6.4 (5.0–7.7)	<0.0001
Circulatory	25.5 (23.2–28.0)	24.0 (20.7–27.3)	21.1 (17.8–24.5)	20.8 (17.5–24.1)	19.9 (16.5–23.3)	0.0003
Respiratory	22.5 (18.8–26.2)	21.3 (17.1–25.5)	23.2 (18.2–28.2)	21.3 (15.9–26.6)	22.0 (16.4–27.6)	0.8604
Perinatal	3.7 (3.2–4.1)	2.3 (1.6–3.0)	2.3 (1.6–3.0)	2.4 (1.7–3.1)	2.3 (1.6–3.0)	0.0036
Congenital	1.2 (0.9–1.5)	1.5 (1.1–1.8)	1.0 (0.6–1.4)	1.2 (0.8–1.6)	1.2 (0.8–1.5)	0.4684

^1^ Adjusted for: age; proportion Australian-born; employed full-time; Indigenous; white collar (managerial, administrative, professional); weighted average of median household income; and weighted average of mean household size. CI: confidence interval.

**Table 2:** Adjusted and unadjusted male all-age hospitalisation rates (per 1000 persons), 95% CI, and significance for trend across gas well development activity quintiles in a coal seam gas area in Queensland, Australia ^1^.

	Very Low (Jan. 1995–May 1998)	Low (Jun. 1998–Sep. 2001)	Medium (Oct. 2001–Feb. 2005)	High (Mar. 2005–Jul. 2008)	Intense (Aug. 2008–Dec. 2011)	*p*-Value for Linear Trend
*Adjusted*						
All-cause	294.2 (263.6–324.8)	316.8 (295.6–337.9)	320.0 (292.0–348.0)	335.5 (300.7–370.3)	335.4 (297.7–373.2)	0.0339
Blood/immune	3.7 (1.4–5.9)	3.4 (2.2–4.5)	3.7 (1.9–5.4)	4.3 (2.1–6.6)	6.1 (3.8–8.3)	0.0679
Circulatory	33.9 (28.7–39.1)	31.7 (28.7–34.6)	26.0 (21.7–30.2)	25.7 (20.2–31.2)	24.3 (18.6–29.9)	0.0010
Respiratory	28.4 (21.6–35.2)	28.7 (23.8–33.6)	30.5 (24.1–36.9)	27.7 (20.0–35.4)	26.3 (17.8–34.8)	0.6931
Perinatal	3.4 (1.8–5.1)	1.5 (0.7–2.4)	1.2 (−0.1–2.5)	1.0 (−0.7–2.7)	1.4 (−0.3–3.0)	0.0089
Congenital	3.1 (2.2–4.1)	2.8 (2.3–3.3)	2.6 (1.8–3.4)	2.8 (1.9–3.8)	2.4 (1.4–3.4)	0.0528
*Unadjusted*						
All-cause	298.6 (281.2–316.0)	322.4 (300.8–344.0)	321.7 (297.4–346.1)	341.4 (316.4–366.4)	346.0 (320.3–371.8)	0.0004
Blood/immune	2.7 (1.9–3.5)	2.4 (1.3–3.6)	2.4 (1.3–3.6)	3.6 (2.4–4.7)	5.4 (4.2–6.5)	<0.0001
Circulatory	32.6 (30.3–34.8)	30.5 (27.4–33.6)	25.8 (22.6–28.9)	27.9 (24.8–31.1)	27.4 (24.2–30.6)	0.0020
Respiratory	25.2 (21.1–29.3)	25.6 (20.7–30.4)	27.4 (21.7–33.0)	25.3 (19.4–31.2)	24.2 (18.1–30.4)	0.8262
Perinatal	4.5 (3.9–5.1)	2.6 (1.8–3.4)	2.7 (1.8–3.5)	2.3 (1.4–3.1)	2.7 (1.9–3.6)	0.0009
Congenital	2.3 (2.0–2.7)	2.0 (1.5–2.5)	1.7 (1.2–2.2)	2.1 (1.6–2.6)	1.6 (1.1–2.1)	0.0267

^1^ Adjusted for: age; proportion Australian-born; employed full-time; Indigenous; white collar (managerial, administrative, professional); weighted average of median household income; and weighted average of mean household size. CI: confidence interval.

## Supplementary Materials

The following are available online at www.mdpi.com/1660-4601/14/5/540/s1, Table S1. Adjusted and unadjusted female hospitalisation rates (per 1000 persons), 95% CI, and significance for trend across gas well development activity quintiles in a coal seam gas area in Queensland, Australia.^1,2^; Table S2. Adjusted and unadjusted male hospitalisation rates (per 1000 persons), 95% CI, and significance for trend across gas well development activity quintiles in a coal seam gas area in Queensland, Australia.^1,2^
