# Supplementary material for: Is Increasing Coal Seam Gas Well Development Activity Associated with Increasing Hospitalisation Rates in Queensland, Australia? An Exploratory Analysis 1995–2011
Source: Int J Environ Res Public Health. 2017 May 18;14(5):540. doi: 10.3390/ijerph14050540 (PMC5451990; doi:10.3390/ijerph14050540)
Supplement: Supplementary file 1 [file ijerph-14-00540-s001.pdf]

Table S1. Adjusted and unadjusted **female** hospitalisation rates (per 1000 persons), 95% CI, and significance for trend across gas well development activity quintiles in a coal seam gas area in Queensland, Australia.[1,2]

	**Very low (Jan 95 – May 98)**	**Low (Jun 98 – Sep 01)**	**Medium (Oct 01 – Feb 05)**	**High (Mar 05 – Jul 08)**	**Intense (Aug 08 – Dec 11)**	***p*-value for linear trend**
Adjusted						
Infectious disease	4.9 (1.9-7.9)	6.1 (4.5-7.8)	9.0 (6.6-11.4)	10.0 (6.8-13.1)	12.6 (9.4-15.8)	0.0000
Neoplasms	25.7 (20.3-31.1)	28.9 (26.1-31.8)	30.3 (26.0-34.7)	29.2 (23.6-34.7)	29.3 (23.7-35.0)	0.0942
Endocrine	3.8 (1.3-6.4)	5.0 (3.7-6.4)	8.9 (6.9-10.9)	9.9 (7.3-12.5)	10.9 (8.2-13.5)	0.0000
Mental disorders	10.4 (6.7-14.0)	12.1 (10.3-13.9)	13.8 (10.9-16.7)	9.5 (5.9-13.2)	8.6 (4.9-12.3)	0.9058
Nervous system	6.0 (3.4-8.6)	7.6 (6.3-8.9)	7.6 (5.5-9.6)	8.1 (5.5-10.7)	11.2 (8.6-13.8)	0.0000
Eye	7.0 (3.4-10.5)	6.9 (4.7-9.0)	7.5 (4.5-10.4)	9.4 (5.6-13.2)	10.2 (6.2-14.1)	0.1552
Ear	2.4 (1.5-3.4)	2.2 (1.7-2.7)	2.4 (1.6-3.1)	2.6 (1.6-3.6)	3.4 (2.4-4.4)	0.0798
Digestive	42.4 (36.7-48.2)	43.6 (40.1-47.1)	39.2 (34.4-44.0)	42.3 (36.1-48.4)	38.8 (32.4-45.3)	0.2640
Skin	4.2 (2.5-5.9)	3.8 (2.9-4.7)	5.4 (4.1-6.8)	4.6 (2.8-6.3)	5.3 (3.5-7.0)	0.2386
Musculoskeletal	16.4 (13.2-20.0)	16.9 (15.3-18.5)	16.9 (14.3-19.4)	18.0 (14.8-21.2)	19.2 (16.0-22.5)	0.0648
Genitourinary	21.3 (17.8-24.7)	19.1 (17.3-21.0)	22.5 (19.7-25.3)	24.9 (21.4-28.5)	25.8 (22.2-29.4)	0.1234
Pregnancy	45.4 (38.4-52.4)	46.3 (42.6-50.0)	50.7 (45.1-56.3)	55.0 (47.8-62.2)	51.7 (44.4-59.0)	0.2191
Symptoms	22.4 (18.5-26.3)	21.8 (19.7-23.9)	21.0 (17.8-24.1)	19.6 (15.6-23.6)	22.4 (18.3-26.5)	0.8043
Injuries	18.8 (14.3-23.4)	21.0 (18.6-23.4)	21.6 (17.9-25.2)	25.5 (20.8-30.1)	26.9 (22.2-31.6)	0.0008
Unadjusted						
Infectious disease	5.4 (4.2-6.7)	6.6 (4.9-8.3)	7.2 (5.5-9.0)	6.9 (5.2-8.7)	9.6 (7.8-11.3)	<0.0001
Neoplasms	19.3 (17.2-21.4)	22.6 (19.6-25.5)	24.6 (21.5-27.6)	25.2 (22.2-28.2)	24.8 (21.8-27.9)	0.0002
Endocrine	3.9 (2.9-4.9)	5.1 (3.7-6.4)	7.5 (6.1-9.0)	8.5 (7.1-9.9)	9.7 (8.3-11.2)	<0.0001
Mental disorders	9.4 (8.1-10.7)	11.1 (9.3-12.9)	13.8 (11.9-15.6)	9.4 (7.5-11.2)	8.0 (6.1-9.9)	0.2001
Nervous system	6.6 (5.6-7.5)	8.1 (6.8-9.5)	8.0 (6.6-9.3)	9.4 (8.1-10.7)	13.0 (11.7-14.3)	<0.0001
Eye	7.8 (6.2-9.5)	7.9 (5.7-10.1)	8.2 (5.9-10.5)	11.1 (8.8-13.4)	13.0 (11.7-14.3)	<0.0001
Ear	2.4 (2.1-2.8)	2.2 (1.6-2.7)	2.1 (1.5-2.6)	2.0 (1.5-2.6)	2.8 (2.3-3.3)	0.3153
Digestive	42.7 (40.1-45.3)	44.1 (40.7-47.6)	40.8 (37.1-44.5)	45.5 (41.8-49.2)	42.8 (39.1-46.6)	0.7362
Skin	4.9 (4.3-5.6)	4.6 (3.7-5.5)	5.4 (4.5-6.3)	5.0 (4.0-5.9)	6.2 (5.3-7.1)	0.0107
Musculoskeletal	16.7 (15.6-17.8)	17.3 (15.7-18.9)	16.0 (14.4-17.6)	17.8 (16.2-19.4)	19.7 (18.1-21.3)	0.0017
Genitourinary	25.5 (24.0-27.1)	23.4 (21.2-25.5)	22.9 (20.7-25.0)	21.8 (19.6-24.0)	22.7 (20.5-24.9)	0.0086
Pregnancy	49.5 (46.5-52.4)	50.6 (46.4-54.7)	51.4 (47.2-55.6)	59.2 (55.0-63.4)	59.1 (54.9-63.4)	<0.0001
Symptoms	23.1 (21.3-24.9)	22.9 (20.4-25.3)	23.5 (21.0-26.1)	25.6 (23.1-28.2)	29.9 (27.4-32.5)	<0.0001
Injuries	26.2 (24.2-28.1)	28.3 (25.6-31.0)	26.1 (23.3-28.9)	27.3 (24.6-30.1)	29.4 (26.6-32.1)	0.0953

[1] Adjusted for: age; proportion Australian-born; employed full-time; Indigenous; white collar (managerial, administrative, professional); weighted average of median household income; and weighted average of mean household size.

[2] ICD code ranges: A00-B99 = *'Certain infectious and parasitic diseases'*; C00-D48 = *'Neoplasms'*; D50-D89 = *'Diseases of the blood and blood-forming organs and certain disorders involving the immune mechanism'*; E00-E90 = *'Endocrine, nutritional and metabolic diseases'*; F00-F99 = *'Mental and behavioural disorders'*; G00-G99 = *'Diseases of the nervous system'*; H00-H59 = *'Diseases of the eye and adnexa'*; H60-H95 = *'Diseases of the ear and mastoid process'*; I00-I99 = *'Diseases of the circulatory system'*; J00-J99 = *'Diseases of the respiratory system'*; K00-K93 = *'Diseases of the digestive system'*; L00-L99 = *'Diseases of the skin and subcutaneous tissue'*; M00-M99 = *'Diseases of the musculoskeletal system and connective tissue'*; N00-N99 = *'Diseases of the genitourinary system'*; O00-O99 = *'Pregnancy, childbirth and the puerperium'*; P00-P96 = *'Certain conditions originating in the perinatal period'*; Q00-Q99 = *'Congenital malformations, deformations and chromosomal abnormalities'*; R00-R99 = *'Symptoms, signs and abnormal clinical and laboratory findings, not elsewhere classified'*; and S00-T98 = *'Injury, poisoning and certain other consequences of external causes'*.

Table S2. Adjusted and unadjusted **male** hospitalisation rates (per 1000 persons), 95% CI, and significance for trend across gas well development activity quintiles in a coal seam gas area in Queensland, Australia.[1,2]

	**Very low (Jan 95 – May 98)**	**Low (Jun 98 – Sep 01)**	**Medium (Oct 01 – Feb 05)**	**High (Mar 05 – Jul 08)**	**Intense (Aug 08 – Dec 11)**	***p*-value for linear trend**
Adjusted						
Infectious disease	5.8 (3.3-8.4)	6.9 (5.4-8.5)	10.6 (8.4-12.8)	10.7 (7.9-13.5)	10.6 (7.7-13.5)	0.0020
Neoplasms	21.1 (16.0-26.3)	21.6 (18.7-24.5)	24.0 (19.8-28.3)	28.7 (23.3-34.2)	28.4 (22.7-34.0)	0.0409
Endocrine	4.5 (3.1-6.0)	5.2 (4.5-5.8)	5.0 (3.8-6.1)	6.3 (4.9-7.7)	6.2 (4.7-7.6)	0.0353
Mental disorders	10.3 (7.1-13.5)	11.0 (9.4-12.7)	10.6 (8.1-13.1)	14.7 (11.5-17.9)	13.4 (10.1-16.6)	0.2341
Nervous system	4.0 (1.0-7.0)	7.4 (6.0-8.8)	7.1 (4.7-9.4)	9.4 (6.5-12.4)	12.0 (9.1-14.9)	<0.0001
Eye	6.5 (3.6-9.5)	6.4 (4.6-8.1)	6.8 (4.3-9.3)	7.6 (4.4-10.7)	9.6 (6.3-12.9)	0.0873
Ear	2.4 (1.3-3.4)	2.0 (1.4-2.6)	2.3 (1.4-3.1)	1.9 (0.8-3.0)	1.9 (0.8-3.0)	0.3671
Digestive	40.1 (35.2-45.0)	44.1 (41.1-47.1)	42.6 (38.5-46.7)	42.0 (36.8-47.3)	37.4 (31.8-42.9)	0.4980
Skin	4.2 (1.9-6.5)	5.7 (4.6-6.8)	6.6 (4.8-8.4)	6.6 (4.3-8.9)	6.6 (4.4-8.9)	0.0138
Musculoskeletal	18.5 (14.7-22.3)	20.1 (18.1-22.0)	19.0 (16.0-22.0)	17.5 (13.7-21.4)	17.2 (13.3-21.1)	0.8905
Genitourinary	19.8 (16.1-23.6)	17.2 (15.3-19.2)	18.4 (15.4-21.4)	23.1 (19.3-27.0)	19.8 (15.9-23.7)	0.3774
Symptoms	19.1 (15.1-23.0)	22.6 (20.5-24.6)	18.5 (15.3-21.7)	18.8 (14.7-22.8)	22.4 (18.3-26.4)	0.0148
Injuries	28.6 (21.6-35.6)	36.0 (32.5-39.4)	38.2 (32.7-43.8)	47.0 (40.0-54.1)	37.7 (30.6-44.8)	0.1439
Unadjusted						
Infectious disease	5.3 (4.1-6.6)	6.3 (4.6-7.9)	7.9 (6.1-9.7)	7.3 (5.6-9.1)	7.3 (5.5-9.1)	0.0191
Neoplasms	22.0 (19.9-24.1)	22.6 (19.6-25.5)	24.4 (21.4-27.5)	30.1 (27.0-33.1)	30.5 (27.5-33.6)	<0.0001
Endocrine	3.2 (2.7-3.7)	3.8 (3.1-4.5)	4.0 (3.3-4.7)	6.1 (5.4-6.8)	6.0 (5.3-6.7)	<0.0001
Mental disorders	8.4 (7.1-9.8)	9.0 (7.1-10.8)	9.3 (7.5-11.2)	11.7 (9.8-13.5)	8.9 (7.0-10.8)	0.1185
Nervous system	7.2 (6.1-8.2)	10.6 (9.1-12.0)	10.0 (8.6-11.5)	11.1 (9.7-12.6)	13.8 (12.4-15.3)	<0.0001
Eye	6.6 (5.3-8.0)	6.6 (4.8-8.4)	6.8 (4.9-8.7)	8.5 (6.6-10.3)	11.1 (9.2-13.0)	<0.0001
Ear	2.6 (2.2-3.1)	2.3 (1.7-2.9)	2.3 (1.7-2.9)	2.3 (1.7-2.9)	2.7 (2.1-3.3)	0.8764
Digestive	40.0 (37.2-41.8)	43.7 (40.6-46.7)	40.8 (37.5-44.0)	41.5 (38.2-44.8)	38.0 (34.7-41.3)	0.2840
Skin	5.4 (4.6-6.2)	6.8 (5.7-7.9)	7.4 (6.3-8.6)	7.1 (6.0-8.2)	7.3 (6.2-8.4)	0.0025
Musculoskeletal	19.3 (17.8-20.7)	20.9 (18.9-22.9)	19.6 (17.6-21.7)	19.7 (17.7-21.7)	20.3 (18.2-22.3)	0.7329
Genitourinary	16.9 (15.4-18.4)	14.2 (12.1-16.3)	14.0 (11.9-16.2)	18.0 (15.9-20.2)	14.2 (12.0-16.4)	0.7291
Symptoms	19.8 (18.3-21.3)	23.3 (21.2-25.4)	21.4 (19.3-23.6)	22.9 (20.8-25.0)	26.7 (24.5-28.8)	<0.0001
Injuries	44.1 (41.0-47.3)	51.2 (46.7-55.6)	45.9 (41.3-50.4)	48.2 (43.7-52.7)	41.0 (36.4-45.5)	0.1737

[1] Adjusted for: age; proportion Australian-born; employed full-time; Indigenous; white collar (managerial, administrative, professional); weighted average of median household income; and weighted average of mean household size.

[2] ICD code ranges: A00-B99 = *'Certain infectious and parasitic diseases'*; C00-D48 = *'Neoplasms'*; D50-D89 = *'Diseases of the blood and blood-forming organs and certain disorders involving the immune mechanism'*; E00-E90 = *'Endocrine, nutritional and metabolic diseases'*; F00-F99 = *'Mental and behavioural disorders'*; G00-G99 = *'Diseases of the nervous system'*; H00-H59 = *'Diseases of the eye and adnexa'*; H60-H95 = *'Diseases of the ear and mastoid process'*; I00-I99 = *'Diseases of the circulatory system'*; J00-J99 = *'Diseases of the respiratory system'*; K00-K93 = *'Diseases of the digestive system'*; L00-L99 = *'Diseases of the skin and subcutaneous tissue'*; M00-M99 = *'Diseases of the musculoskeletal system and connective tissue'*; N00-N99 = *'Diseases of the genitourinary system'*; O00-O99 = *'Pregnancy, childbirth and the puerperium'*; P00-P96 = *'Certain conditions originating in the perinatal period'*; Q00-Q99 = *'Congenital malformations, deformations and chromosomal abnormalities'*; R00-R99 = *'Symptoms, signs and abnormal clinical and laboratory findings, not elsewhere classified'*; and S00-T98 = *'Injury, poisoning and certain other consequences of external causes'*.
